# Impact of the COVID-19 pandemic on therapy compliance and lifestyle factors of patients with growth hormone deficiency

**DOI:** 10.15537/smj.2022.43.4.20210877

**Published:** 2022-04

**Authors:** Sulafa T. Sindi, Njood W. Nazer, Abdulmoein E. AlAgha

**Affiliations:** *From the Department of Pediatrics (Sindi), King Abdulaziz University Hospital; from the Department of Pediatrics (AlAgha), Faculty of Medicine, King Abdulaziz University, and from the Department of Pediatrics (Nazer), King Fahad Armed Forces Hospital, Jeddah, Kingdom of Saudi Arabia.*

**Keywords:** growth hormone, adherence, COVID-19, lifestyle factors

## Abstract

**Objectives:**

To recognize and assess treatment compliance in children and adolescents with growth hormone deficiency during the cronavirus disease-2019 (COVID-19) pandemic along with other lifestyle factors that might have been affected by the pandemic, such as diet, physical activity, sleep pattern, and screen time.

**Methods:**

This descriptive, cross-sectional study was carried out between March 2020-2021 at King Abdulaziz University Hospital, Jeddah, Saudi Arabia. Data were collected through clinical interview questions.

**Results:**

The total sample size was 130 patients, 54 (41.5%) of whom were males and 76 (58.5%) were females. The mean age of the patients was 12.56±3.44 years. Comparisons of before and during the COVID-19 pandemic revealed significant changes in growth hormone therapy compliance (*p*=0.007), dietary changes (*p*=0.002) with an increase in vegetables and fruit consumption, a significant decrease in physical activity time (*p*<0.001), an increase in sleep time (*p*<0.001), and screen time (*p*<0.001).

**Conclusion:**

The COVID-19 crisis had an impact on growth hormone therapy compliance, with a significant impact on other lifestyle factors such as dietary habits, physical activity, sleep time, and leisure screen time.

**G**rowth hormone is principally involved in linear growth regulation.^1^ Growth hormone deficiency (GHD) is characterized by either the partial reduction or a total absence of GH production.^1^

Patients with GHD undergo treatment with recombinant human GH (rhGH) for a specific period that may last until they achieve complete growth.^2^ Thus, optimal and satisfying growth response achievement during rhGH therapy is affected by multiple factors, and most importantly, by compliance to GH therapy.^3^ According to the World Health Organization (WHO), compliance is the extent to which a person’s behavior regarding medication compliance corresponds with agreed recommendations by the primary healthcare provider.^4^ Non-compliance is when the individual undergoing treatment misses more than one injection per week.^5^ Growth hormone therapy is a safe and effective treatment for GHD; it involves the injection of GH daily for a specified period, and compliance is important to ensure optimal therapy outcome.^5^ Compliance to GH therapy may be affected by multiple factors, including misperceptions regarding the consequences of missed GH doses, and inadequate contact with health-care providers, which may in turn affect the efficacy of the therapy. Other factors affecting GH efficacy include sleep deprivation, lack of exercise, a high-fat diet, and financial burden.^6,7^

Compliance to a daily injection regimen for GH therapy may have been affected by the mandatory lockdowns in response to the coronavirus disease-2019 (COVID-19) pandemic.^8,9^ Failure to comply with treatment as a result of the COVID-19 health system disruptions has led to reduced physical health and treatment effectiveness.^10^ Among possible reasons for noncompliance are missed appointments with healthcare providers, forgotten daily injections, a lack of awareness of the importance of therapy, the inability to pay for injections, long course of treatment, and the refusal of the patient to submit to therapy. In this study, we aimed to clarify the impact of COVID-19 on GH therapy compliance and to assess lifestyle factors that might have been affected by the pandemic.

## Methods

This cross-sectional study assessed patients with GHD. Patients of any gender and ethnicity aged 1-20 years were included. Patients or their guardians provided consent to participate. The study was approved by the Ethics Committee of King Abdulaziz University Hospital, Jeddah, Saudi Arabia.

Data was collected between March 2020-2021 through a clinical interview. Questions that facilitated the analysis of the data included: demographic information; non-compliance to growth hormone therapy, including the number of missed doses per week; and lifestyle factors that may have been affected by COVID-19 including dietary habits, physical activity, sleep pattern, and screen time.

Patients aged 1-20 years old, those who followed up regularly prior to COVID-19, and those undergoing GH therapy were included, while patients aged <1 and >20 years old, those who did not follow up regularly prior to COVID-19, and those who were not undergoing GH therapy were excluded.

Sample size was calculated in this study using the Robert Mason equation which revealed a sample size of 97. This study included 130 out of 200 patients initially recruited after applying the inclusion and exclusion criteria.

### Statistical analysis

Statistical Package for the Social Sciences, version 20.0 (IBM Corp., Armonk, NY, USA)was used. Simple frequency tables, cross-tabulations, and percentages were calculated. The Chi-square test was used to describe the relationship between 2 categorical variables. A *p*-value of <0.05 was considered significant.

## Results

The study included 54 (41.5%) males and 76 (58.5%) females. The mean age of the study participants was 12.56±3.44 years and the mean age at the initiation of rhGH therapy was 8.46±3.15 years.

Analysis of growth hormone therapy compliance revealed that 92% complied to their rhGH therapy regimen during the COVID-19 pandemic, while 8% failed to comply (*p*=0.007). Table 1 shows the causes of non-compliance.

**Table 1:** - Demographic data (N=130).

Parameter	n (%)
***Gender***	
Male	54 (41.5)
Female	76 (58.5)
Age (years), mean±SD	12.56±3.44
Age (years) at the initiation of rhGH therapy, mean±SD	8.46±3.15

SD: standard deviation, rhGH: recombinant human growth hormone

Dietary habits were greatly impacted by the COVID-19 pandemic. The percentage of participants (54.6%) who consumed a high-protein diet prior to the COVID-19 pandemic increased to 60% during the pandemic. Fast food consumption among participants decreased from 53.1% before COVID-19 to 46.2% during the pandemic (*p*=0.028). The number of participants who consumed desserts and sweetened drinks before the COVID-19 pandemic, increased from 49.2% to 51.5% during the pandemic (*p*=0.407), while vegetable and fruit intake also significantly increased among the study population prior to and during the COVID-19 pandemic (*p*=0.002).

Another factor that was significantly affected by the COVID-19 pandemic was physical activity (*p*=0.001). In this study population, 29.8% who carried out physical activity daily for <30 minutes and 18.2% who performed physical activity daily for >30 minutes prior to the COVID-19 pandemic, stopped exercising during the pandemic. Of those who did not carry out any physical activity prior to the pandemic, 79.5% continued not to do so, while 17.9% started to exercise for <30 minutes and 2.6% exercised for >30 minutes. Nevertheless, 63.8% of patients performing physical activity <30 minutes daily prior to the COVID-19 pandemic, continued to have the same duration of physical activity in contrast to a 6.4% of patients who increased their physical activity time to >30 minutes. Of those who used to exercise for >30 minutes before the pandemic, 61.4% continued to do so, while 20.5% reduced the duration of their exercise to <30 minutes.

**Figure 1:**
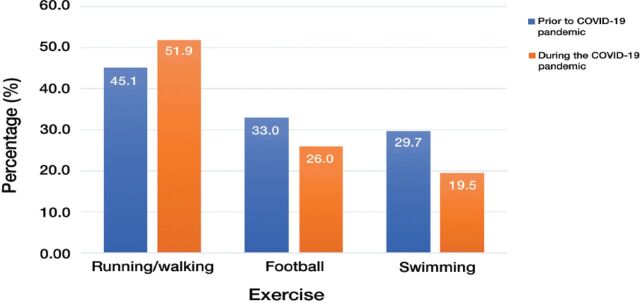
- Comparison of types of exercise carried out prior to versus during the coronavirus disease-2019 (COVID-19) pandemic.

Figure 1 provides additional information regarding exercise types before and during COVID-19 stratified by the commonest type.

Among those who had <8 hours of sleep before the COVID-19 pandemic, 64.6% continued to sleep for <8 hours during the pandemic, while 35.4% increased their sleep duration to 8 hours (*p*<0.001). Among those who slept for >8 hours before the COVID-19 pandemic, sleep duration decreased to <8 hours in 6.2%, while 93.8% continued to sleep for >8 hours during the pandemic.

The assessment of leisure screen time during the COVID-19 pandemic showed that the percentage of patients who used to spend <2 hours on screen time (38.2%) continued to have the same duration of screen time during the pandemic. Meanwhile, 23.6% of total participants who spent <2 hours prior to pandemic, have increased their screen time to 2-4 hours and 38.2% had >4 hours of screen time during the COVID-19 pandemic (*p*<0.001). Less than a third (31.7%) of patients who spent 2-4 hours on screen time prior to the pandemic, continued to do so during the pandemic, while only 2.4% had less leisure time (<2 hours) during the pandemic, and 65.9% spent >4 hours of leisure screen time during the pandemic. However, of those who used to spend >4 hours on screen time prior to the pandemic, 94.1% continued to do so during the pandemic crisis, while 5.9% decreased their leisure time to 2-4 hours.

## Discussion

**Figure 2.**
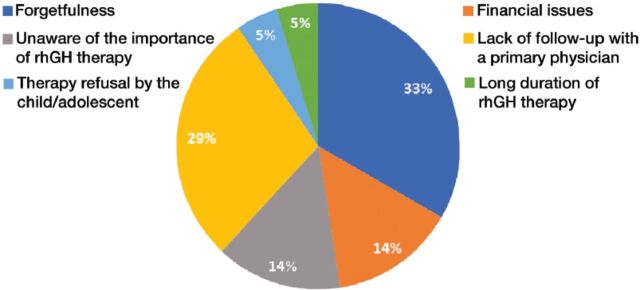
Factors that negatively affect compliance to recombinant human growth hormone (rhGH) therapy.

This study revealed that therapy compliance was affected by the COVID-19 pandemic. Among those participants who were compliant to medication before the pandemic, 92% continued to be compliant with rhGH treatment during the pandemic, despite the huge impact that COVID-19 had on the health system. Reasons for non-compliance are shown in Figure 2, the most common reason being forgetfulness (53.8%). In a prior study carried out in Italy, the most common reasons for non-compliance included being away from home (33.3%), forgetfulness (24.6%), not feeling well (12.9%), and pain (10.3%).^7^

The COVID-19 pandemic is affecting and changing family dynamics, including but not limited to, dietary habits.^11^ A study that included 1003 parents of children aged 0-12 years reported that 61.5% experienced changes in daily routine and eating habits. These resulted in an increase in consumption of foods that consisted mainly of carbohydrate, meat, and sweetened drink intake.^11,12^ In this study, protein-rich foods were the most frequently consumed before the pandemic, and consumption increased during the pandemic while fast food intake was noted to have decreased during the pandemic. Participants reported consuming more desserts and sweetened drinks during the pandemic, with a significant increase in participants consuming vegetables and fruit during the pandemic.

The WHO guidance stated that children and adolescents aged 5-17 years should participate in at least 60 minutes of moderate to vigorous physical activity per day.^13^ Levels of physical activity decreased in people of all ages during the COVID-19 pandemic. In this study, the decreased duration of physical activity reported prior to versus during the pandemic was significant. Those who exercised daily for <30 minutes and >30 minutes before the pandemic, stopped exercising during the pandemic, while a small proportion who had never exercised before the pandemic started to do so. Regarding the types of exercise, this study’s findings revealed that the participants most frequently participated in running/walking rather than in soccer and swimming.

A study carried out in Italy determined the impact of COVID-19 on sleep patterns.^14^ The study revealed that 67.4% of participants suffered from sleep disturbance. In comparison, the present study showed that <50% of those who slept for <8 hours before the pandemic slept for >8 hours during the pandemic, and 64.6% continued to have <8 hours of sleep. The majority of participants who slept for >8 hours before the pandemic continued the same sleeping pattern, while a small proportion slept <8 hours.

The recommended screen time for children and adolescents, as per the American Academy of Pediatrics, is ≤2 hours.^15^ A prior study revealed that screen time increased throughout lockdowns associated with the COVID-19 pandemic.^15^ The cohort study demonstrated that an average of 6 hours was spent on leisure screen time during the pandemic, which corresponded to a 94% increase. This study concluded that 23.6 % of patients who had <2 hours of screen time prior to the pandemic increased their screen time to 2-4 hours during the pandemic, while 38.2% significantly increased their screen time to >4 hours.

### Study limitations

First, results of this study were generated from a single center, thus a need for multi-center study for a more generalized data interpretation. A small sample size of patients was included. Thus, findings could not be generalized for the entire Saudi Arabian population. Second, the use of convenience sampling could have aggravated the risk of selection bias. Third, the growth velocity was not assessed due to the lack of information provided by the guardian.

In conclusion, COVID-19 pandemic had a significant impact on the healthcare system, and therefore, on GH therapy compliance. In addition, it has impacted several lifestyle factors including but not limited to dietary habits, physical activity, sleep patterns, and leisure screen time. Thus, more data should be further studied in order to raise more awareness in regard to medication compliance in such a pandemic and to enhance lifestyle factors accordingly.
